# Intestinal antimicrobial peptides during homeostasis, infection, and disease

**DOI:** 10.3389/fimmu.2012.00310

**Published:** 2012-10-09

**Authors:** Luciana R. Muniz, Camille Knosp, Garabet Yeretssian

**Affiliations:** Department of Medicine, Immunology Institute, Mount Sinai School of MedicineNew York, NY, USA

**Keywords:** antimicrobial peptides, enteric pathogens, homeostasis, inflammatory bowel disease, innate immunity, intestine, NOD-like receptors, Toll-like receptors

## Abstract

Antimicrobial peptides (AMPs), including defensins and cathelicidins, constitute an arsenal of innate regulators of paramount importance in the gut. The intestinal epithelium is exposed to myriad of enteric pathogens and these endogenous peptides are essential to fend off microbes and protect against infections. It is becoming increasingly evident that AMPs shape the composition of the commensal microbiota and help maintain intestinal homeostasis. They contribute to innate immunity, hence playing important functions in health and disease. AMP expression is tightly controlled by the engagement of pattern recognition receptors (PRRs) and their impairment is linked to abnormal host responses to infection and inflammatory bowel diseases (IBD). In this review, we provide an overview of the mucosal immune barriers and the intricate crosstalk between the host and the microbiota during homeostasis. We focus on the AMPs and pay particular attention to how PRRs promote their secretion in the intestine. Furthermore, we discuss their production and main functions in three different scenarios, at steady state, throughout infection with enteric pathogens and IBD.

## INTRODUCTION

The human gut is colonized by trillions of indigenous microorganisms that constitute one of the most complex microbial communities on earth (Backhed et al., 2005; Gill et al., 2006; Turnbaugh et al., 2007). These microorganisms live in symbiotic and mutualistic relationship with the host, and thus make up the microbiome that is essential for mediating physiology, metabolism and host immune responses. The gut mucosa is exposed to myriad food and microbial antigens that require different types of responses (tolerance, suppression, or active immunity) determined by whether the antigen is likely to be beneficial or detrimental to the host (Mowat, 2003). The gastrointestinal (GI) tract is also a major entry point for enteric pathogens usually co-ingested with food and the organism has developed multilevel defense mechanisms to fight against this challenge.

**FIGURE 1:**
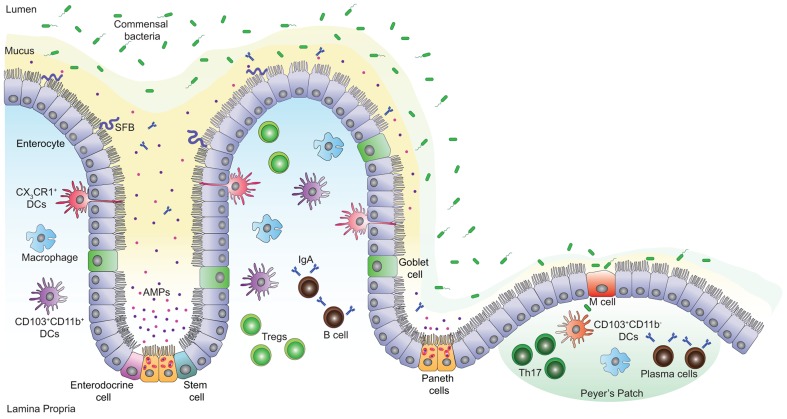
**Intestinal mucosal surface at the steady state.** The intestinal epithelial barrier is a highly organized mucosal surface that prevents the entry of microbes into the lamina propria. The epithelium is constituted of a single layer of intestinal epithelial cells (IECs) covered by a stratified mucus layer. Unlike the outer mucus layer that is colonized by commensal species, the inner mucus layer is mostly devoid of bacteria; it contains Immunoglobulin A (IgA) and antimicrobial peptides (AMPs) that avert the commensal species from interacting with the surface of the IECs. Yet, few opportunistic bacteria such as segmented filamentous bacteria (SFB) can breach the mucus barrier and enter in contact with the IECs. There are five IEC lineages derived from epithelial stem cells that proliferate and give rise to daughter cells. These cells include enterocytes, mucus-producing goblet cells, hormone-producing enteroendocrine cells, AMP-producing Paneth cells at the base of the crypts and finally, M cells that sample antigens from the intestinal lumen in order to present them to nearby immune cells. A high number of T cells, macrophages, IgA secreting B and plasma cells are present in the lamina propria and the Peyer’s patches. In addition, CD103^+^CD11b^-^ and CD103^+^CD11b^+^ dendritic cells (DCs) promote the development of T helper (Th17) and T regulatory (Treg) cells respectively, while CX_3_CR1^+^ DCs sample the lumen antigenic content.

The intestinal mucosal surface acts as a primary barrier against microbial invaders and toxins, whereas commensals are in a dynamic and intimate interaction with the gut epithelium and influence the host cellular and immune responses. Chemical barriers, such as the acidic pH of the stomach and the bile fluid, provide one of the first constitutive defenses of the GI tract. Gastric fluids maintain the stomach pH below 3 limiting infections with pathogenic microbes (Tennant et al., 2008), whereas the bile, particularly bile salts, have bactericidal functions and neutralize enteric bacteria by specifically affecting their DNA and membrane integrity (Begley et al., 2005). Other defense mechanisms in the gut include mechanical and immunological barriers that protect the organism from toxins and infections. The physical barrier is formed by a single layer of epithelial cells covered by a stratified layer of mucus (**Figure 1**). The latter is a mesh-like network of fibers composed of mucins, glycoproteins, lipids, antimicrobial peptides (AMPs), and secretory immunoglobulin A (IgA) that controls the passage of particular molecules (Neutra et al., 1996; McGuckin et al., 2011) and prevents bacteria from reaching the epithelial barrier. The mucus is constituted of two layers: a firm epithelium-associated inner layer or glycocalyx and a viscous outer layer (Sonnenburg et al., 2004; Kelly et al., 2007; Macfarlane and Dillon, 2007; Linden et al., 2008). In the glycocalyx, mucins contain membrane-binding domains that bind and stabilize critical GI trophic factors potentially contributing to epithelial restitution (Corfield et al., 2001). The outer layer, however, contains soluble mucins secreted primarily by goblet cells. The thickness of these two layers varies in each part of the GI tract and is dependent on the presence of commensal bacteria, being thinner where the microbiota is less abundant (Atuma et al., 2001; Robbe et al., 2004). Typically, the inner mucus layer is free of microorganisms and commensals occupy only the outer mucus layer forming a biofilm that allows their growth and prevents their removal during peristalsis (Macfarlane and Dillon, 2007; Johansson et al., 2008).

The intestinal epithelium tightly regulates bidirectional movement of ions, nutrients and cells between the gut lumen and the internal space (Madara, 1990). To ensure these functions and to resist to the microbial passage, intestinal epithelial cells (IECs) are connected to each other with tight junctions, which are formed by integral membrane and cytoskeletal anchor proteins (Shen and Turner, 2006). Moreover, IECs sense microbial-associated molecular patterns (MAMPs), shared between commensals and pathogens, through pattern recognition receptors (PRRs) including Toll-like receptors (TLRs) and NOD-like receptors (NLRs), and elaborate a microorganism-dependent program of intestinal homeostasis and repair (Lavelle et al., 2010; Takeuchi and Akira, 2010; Yeretssian, 2012). This promotes proliferation of epithelial cells, secretion of IgA into the gut lumen and expression of innate immune effector molecules, namely AMPs that likewise have a key homeostatic role in shaping the composition of the microbiota (Salzman et al., 2010). Every immune barrier in the gut contains an array of immune cells that patrol the physical barrier and regulate tissue immunity. Thus, the innate and adaptive immune systems build an additional defense line essential to eliminate microbiota that have bypassed earlier barriers. Enteric pathogens have evolved strategies to either infiltrate or evade the secreted and cellular barriers to infections. Otherwise, defects in the functions of these mucosal barriers alter the gut ecosystem and the microbes gain the potential to invade the host and can lead to intestinal disorders such as inflammatory bowel diseases (IBDs; Sartor, 2008) and irritable bowel syndrome (Parkes et al., 2008), obesity (Ley et al., 2006), diabetes (Wen et al., 2008), etc.

In this review, we describe recent advances in the central emerging role of AMPs in the GI tract. We provide an overview on the dynamic yet intricate interactions between the intestinal epithelial barrier and the lumen microorganisms both commensals and enteric pathogens. We further discuss how innate immune PRRs regulate AMP production during homeostasis, infection, and IBDs.

## MUCOSAL IMMUNE BARRIER AND HOMEOSTASIS

Intestinal homeostasis relies on a tightly regulated crosstalk between commensal bacteria, IECs, and mucosal immune cells. The intestinal epithelium is composed of five cell lineages that include goblet cells, Paneth cells, microfold (M) cells, enteroendocrine cells, and absorptive enterocytes that arise from a pluripotent stem cell progenitor and contribute to barrier integrity through unique and specialized mechanisms (Garrett et al., 2010; **Figure 1**). Intestinal epithelial stem cells proliferate and then differentiate into villous and colonic enterocytes that ensure nutrient adsorption and AMP secretion (Karam, 1999; Bjerknes and Cheng, 2006). Likewise, progenitor IECs differentiate into mucus-secreting goblet cells (Karam, 1999), hormone-producing enteroendocrine cells (Schonhoff et al., 2004), and at the base of the small intestinal crypt, into Paneth cells that produce various AMPs (van Es et al., 2005; Bjerknes and Cheng, 2006). M cells are specialized epithelial cells of the small intestine; together with follicle-associated epithelial cells they overlie the Peyer’s patches and play a major role in sampling the intestinal lumen and presenting its content to nearby immune cells (Miller et al., 2007).

In steady state, the lamina propria and the gut-associated lymphoid tissue (GALT) comprise a large number of T lymphocytes in equal abundance to plasma cells that produce IgA, and also contain macrophages and dendritic cells (DCs; MacDonald and Monteleone, 2005). The complexity of the intestinal mucosal surface is well illustrated by the diversity of cells issued from the lymphoid lineage and the array of cytokines secreted from these cells (Macpherson and Harris, 2004; Cheroutre et al., 2011). In response to lumen antigens, a high fraction of these lymphocytes produce polarized cytokines such as interleukin (IL)-5, IL-13, IL-17, IL-22, and/or interferon (IFN)-γ, which are extensively reduced in germ-free (GF) animals (Hall et al., 2008; Ivanov et al., 2008). The characteristics, functions, and expression of cytokines within the intestine have been recently reviewed (Spits and Di Santo, 2011; Pearson et al., 2012). GF mice exhibit underdeveloped lymphoid tissues with fewer Peyer’s patches, aberrant development and maturation of isolated lymphoid follicles, reduced expression of several AMPs, lower numbers of flora-reactive B cells and lamina propria CD4^+^ T cells; this particularly affects the frequencies of T helper (Th1 and Th17) cells but not regulatory T (Treg) cells (Hooper et al., 2003; Mazmanian et al., 2005; Cash et al., 2006; Bouskra et al., 2008; Hall et al., 2008; Ivanov et al., 2008; Hapfelmeier et al., 2010). Interestingly, these defects can be adjusted by colonization of the mice with a complex microbiota or specific bacterial species that orchestrates T cell responses (Talham et al., 1999; Umesaki et al., 1999; Heczko et al., 2000; Gaboriau-Routhiau et al., 2009; Ivanov et al., 2009). Examples of these specific bacterial species include the segmented filamentous bacteria (SFB) and *Clostridium* species. Colonization of GF mice with SFB enhances the differentiation of Th17 cells, which are required for host resistance against enteric pathogens and drive systemic autoimmunity (Gaboriau-Routhiau et al., 2009; Ivanov et al., 2009; Wu et al., 2010). Proinflammatory Th17 cells are the most abundant cellular source for IL-17 and IL-22 cytokines and they are kept in check by the actions of IL-10 producing lamina propria Treg cells critical for the maintenance of intestinal homeostasis. Indeed, capsular polysaccharide A from the human symbiont *Bacteroides fragilis* beneficially influences the gut immune response by facilitating Foxp3^+^ Treg differentiation and IL-10 production during commensal colonization (Mazmanian et al., 2008; Round and Mazmanian, 2010). Furthermore, colonization of GF mice with a mix of *Clostridium* strains provided an environment rich in transforming-growth factor-β (TGF-β) and promoted Foxp3^+^ Treg cell accumulation and activity in the colon (Atarashi et al., 2011).

Specialized subsets of DCs and macrophages are also present in healthy lamina propria and they differentially induce Th17 and Treg cell responses (Denning et al., 2007; **Figure 1**). Macrophages display an anti-inflammatory profile but are fully capable of killing bacteria whereas DCs are basically proinflammatory (Kelsall, 2008). DCs can be divided into two main populations based on the expression of CX_3_CR1 (CX3C chemokine receptor 1) and CD103 (αE integrin) both of which promise the ability of DCs to control the type and extent of T cell activation (Denning et al., 2007; Schulz et al., 2009; Niess and Adler, 2010). CX_3_CR1^+^ DCs can sense and sample the intestinal lumen through protrusions across the epithelial barrier and their expansion seems to depend on the presence of microbiota (Niess et al., 2005; Niess and Adler, 2010). Indeed, CX_3_CR1^+^ DCs help Th17 cell differentiation (Denning et al., 2007; Atarashi et al., 2008; Uematsu et al., 2008); however a fraction of these cells contribute to the expansion of Foxp3^+^ Treg cells during oral tolerance (Hadis et al., 2011). CD103^+^ DCs express high levels of retinoic acid and TGF-β that together favor the induction of Foxp3^+^ Treg cells and drive the differentiation of tolerogenic DCs (Coombes et al., 2007; Sun et al., 2007; Iliev et al., 2009). While immune cells are essential to conserve the balance at the gut mucosal surface, AMPs have a major role in reinforcing this barrier.

## INTESTINAL AMPs AND HOMEOSTASIS

Innate immunity provides the first line of defense against invading microorganisms and confers protection by triggering inflammatory and antimicrobial responses. PRRs and AMPs are evolutionarily conserved effector molecules of the innate immune system within the gut. They help increase IEC potential to avert bacterial uptake, a critical process for maintaining a functional gut immune barrier and homeostasis. The expression of AMPs is tightly regulated by the presence of microorganisms via different mechanisms mainly implicating the activation of PRRs (TLRs and NLRs) in IECs (Cash et al., 2006). Among the various AMPs produced in the GI tract, defensins and cathelicidins constitute the two major classes. Likewise, IECs secrete slightly larger AMP molecules with comparable bactericidal activities.

## DEFENSINS

Defensins are small, cationic peptides containing disulfide bonds necessary to damage the bacterial cell membrane and eradicate bacteria (Ganz, 2003). These small peptides are further classified as α- and β-defensins depending on the position of their disulfide bonds (Ganz, 2003). The α-defensins (also called cryptdins in mice) comprise four human neutrophil peptides (HNPs 1–4) present in neutrophils and two human α-defensins (HD-5 and HD-6) that are mainly made by Paneth cells, with significantly lower expression in the colon than in the small intestine (Porter et al., 2002; Ganz, 2003). Interestingly, HD-5 has the ability like lectins to bind to glycosylated proteins and neutralize bacterial exotoxins (Lehrer et al., 2009). Mice encode 19 cryptdins and six cryptdin-related sequence (CRS) peptides in a single 1-Mb locus with high heterogeneity between mouse strains (Ouellette, 2006; Shanahan et al., 2011). Synthesis of these peptides is mainly limited to Paneth cells of the small intestine. The α-defensins are accumulated in secretory granules of Paneth cells as inactive pro-peptides and need proteolytic cleavage in order to gain antimicrobial activity (Ouellette, 2006). Cryptdins are activated by matrix metalloproteinase 7 (MMP7) within the granules, while HD-5 and HD-6 are processed by trypsin after their release (Ghosh et al., 2002; Porter et al., 2002; Shirafuji et al., 2003). Compared to α-defensins, the relative expression level of β-defensins is more variable throughout the GI tract. Both humans and mice express quite a large number of β-defensins, of which six human β-defensins (hBDs) and five mouse β-defensins (mBDs) have been identified and characterized. β-Defensins are expressed by many types of epithelial cells, including enterocytes (O’Neil et al., 1999; Frye et al., 2000). The hBD1 and its ortholog mBD1 are constitutively expressed within the GI tract and mostly remain stable during inflammation, while hBD2, hBD3, and hBD4 are induced upon pathogen encounter (O’Neil et al., 1999; Krisanaprakornkit et al., 2000; Takahashi et al., 2001).

In addition to their antimicrobial properties against both Gram-negative and Gram-positive bacteria, defensins promote inflammatory innate and adaptive immune reactions and possess effective chemo-attractant activities (Yang et al., 1999). These mediators are known to recruit and activate different leukocyte populations (Oppenheim and Yang, 2005). Among the β-defensins, hBD1–3 and mBD2/3 show chemotactic activity toward immature DCs and memory T cells (Yang et al., 1999). Furthermore, hBD2 is able to induce recruitment of neutrophils and mast cells (Niyonsaba et al., 2002, 2004). HNPs also contribute to adaptive immunity by selectively mobilizing naïve T cells and immature DCs (Yang et al., 2000). HNP1 and HNP2 have been described to attract monocytes (Territo et al., 1989), while HNP1, HNP3, and HD-5 are responsible for the chemoattraction of macrophages and memory T cells (Grigat et al., 2007). Interestingly, some defensins, such as hBD2, have the ability to directly bind to the CC-chemokine receptors CCR6 and CCR2, and are believed to act as “micro-chemokines” capable of recruiting leukocytes to sites of microbial invasion (Yang et al., 1999; Rohrl et al., 2010). Structurally, hBD2 can be considered as a simplified form of murine CCL20. HBD2 bares aspartic acid and leucine residues placed in similar position and orientation as those in the DCCL motif of CCL20 that provides the interaction with CCR6 receptor (Perez-Canadillas et al., 2001). Thus far, the receptors for α-defensins have not been identified, but it has been suggested that HNPs use G_iα_ protein-coupled receptors since their chemotactic effect is blocked by pertussis toxin (Yang et al., 2000).

Besides their role as chemotactic effectors, defensins modulate the maturation and differentiation of leukocytes. The α-defensins HNP1–3 control the maturation of human monocyte-derived DCs and depending on the dose can up-regulate or down-regulate the maturation of these cells (Rodriguez-Garcia et al., 2009). HBD3 induces the expression of the costimulatory molecules CD80, CD86, and CD40 on myeloid DCs and monocytes through TLR1 and TLR2 (Funderburg et al., 2007), whereas mBD2 induces the activation of DCs in a TLR4-dependent manner (Biragyn et al., 2002). Furthermore, mBD14 is able to switch naïve T cells into a regulatory phenotype by inducing the expression of Foxp3 and cytotoxic T-lymphocyte antigen 4 (CTLA-4; Navid et al., 2012). Finally, both HNP1 and hBD1 were shown to be chemotactic for monocyte-derived DCs, they induce the activation of these cells and enhance the induction of pro-inflammatory mediators (Presicce et al., 2009).

## CATHELICIDINS

Cathelicidins constitute another major class of AMPs expressed on the top of colonic crypts (Hase et al., 2002). In humans and mice only one cathelicidin was identified, LL-37/hCAP18 and CRAMP, respectively. LL-37 is a small and linear peptide characterized by a highly conserved N-terminal structure named cathelin (cathepsin L inhibitor), and a variable C-terminal cationic domain that exerts microbicidal effect (Mendez-Samperio, 2010). It possesses a broad bactericidal activity toward both Gram-negative and Gram-positive bacteria (Turner et al., 1998). LL-37 neutralizes lipopolysaccharide (LPS), it has synergistic antibacterial effects with the defensins and presents chemotactic activity for neutrophils, monocytes, and T cells (Agerberth et al., 2000; De et al., 2000; Nagaoka et al., 2000). Similarly to defensins, LL-37 binding to microbes is driven by electrostatic interactions leading to aqueous pore formation at the surface of bacteria and their subsequent lysis (Herce and Garcia, 2007; Mendez-Samperio, 2010; Sochacki et al., 2011). In addition to its microbicidal activity, it was described that LL-37 interacts with the formyl peptide receptor-like 1 (FPRL-1) and through this interaction contributes to innate and adaptive immunity by recruiting granulocytes, monocytes, and T cells to sites of microbial invasion (Agerberth et al., 2000; De et al., 2000).

## OTHER INTESTINAL ANTIMICROBIALS

Antimicrobial C-type lectins have recently been identified in human and mouse Paneth cells (Lasserre et al., 1999; Vaishnava et al., 2008). These proteins simply consist of a carbohydrate recognition domain and a N-terminal signal peptide (Iovanna and Dagorn, 2005). The main human lectin is the regenerating islet-derived protein 3-alpha (RegIIIα), also known as hepatocarcinoma-intestine-pancreas/pancreatic-associated protein (HIP/PAP). The mouse ortholog of human RegIIIα is RegIIIγ (Cash et al., 2006). Following proteolytic activation by trypsin, both lectins exert antimicrobial actions against Gram-positive bacteria through binding to peptidoglycan (Medveczky et al., 2009; Mukherjee et al., 2009; Lehotzky et al., 2010). Unlike other AMPs, the C-type lectins are induced within the intestine in a TLR/MyD88 (myeloid differentiation primary respon- se protein 88)-dependent manner (Cash et al., 2006; Vaishnava et al., 2008).

Various intestinal enzymes such as lysozyme C, secretory phospholipase A2 type IIA (sPLA2) and angiogenin 4 (ANG4) have also been shown to play antimicrobial functions. In humans, lysozyme C is expressed in Paneth cells and abundant in various cell secretions, whereas in mice it is encoded by two separate genes, one expressed in macrophages (lysozyme C type M) and the other in Paneth cells (lysozyme C type P; Cross et al., 1988; Ganz, 2004). Lysozyme C is a glycoside hydrolase that damages Gram-positive bacterial cell walls by cleaving peptidoglycan and rendering bacteria susceptible to disruption by osmotic pressure (Masschalck and Michiels, 2003). sPLA2 is another important antibacterial gene constitutively expressed by Paneth cells and found to rapidly degrade bacterial phospholipids, thereby destroying cell integrity (Qu et al., 1996; Lambeau and Gelb, 2008). In addition to a strong activity against a wide range of bacteria, sPLA2 has pro-inflammatory properties and possibly confers resistance to intestinal tumorigenesis (Cormier et al., 1997; Bidgood et al., 2000; Buckland and Wilton, 2000). ANG4 is an angiogenic protein secreted by mouse Paneth cells into the gut lumen and belongs to a family of RNases with antibacterial and antiviral functions (Hooper et al., 2003; Crabtree et al., 2007). Colonization of GF-mice with conventional microbiota or specifically with *Bacteroides thetaiotaomicron* enhances the production of ANG4 by Paneth cells (Hooper et al., 2003). This enzyme retains ribonuclease activity that makes it effective against various Gram-positive and Gram-negative microorganisms (Stappenbeck et al., 2002; Crabtree et al., 2007). In direct contact with the microbiota, IECs produce antimicrobial proteins with cationic properties including the bactericidal/permeability-increasing protein (BPI) and a variety of chemokines of which CCL20 (also known as MIP-3α; Izadpanah et al., 2001; Canny et al., 2002). In order to control inflammation in the GI tract and eliminate bacteria, BPI has the ability to bind to LPS and neutralize its activity, whereas CCL20 is a potent chemotactic agent for T cell and DC subsets (Elsbach et al., 1994; Izadpanah et al., 2001; Canny et al., 2002; Yang et al., 2003). Moreover, CCL20 possesses strong bactericidal activity, in some cases of greater potency than hBDs (Hoover et al., 2002). Resistin-like molecule β (RELMβ) is an effector molecule produced by goblet cells and involved in maintaining intestinal epithelium barrier function by up-regulating mucin gene expression and preventing bacterial penetration. It is believed that the presence of the microbiota is associated with the production of RELMβ (Artis et al., 2004; Hogan et al., 2006). The expression of AMPs and other molecules with antimicrobial properties in the gut reveal a mechanism whereby intestinal commensal bacteria influence gut microbial ecology and shape innate immunity.

## PRRs IN PROMOTING AMP SECRETION

**FIGURE 2:**
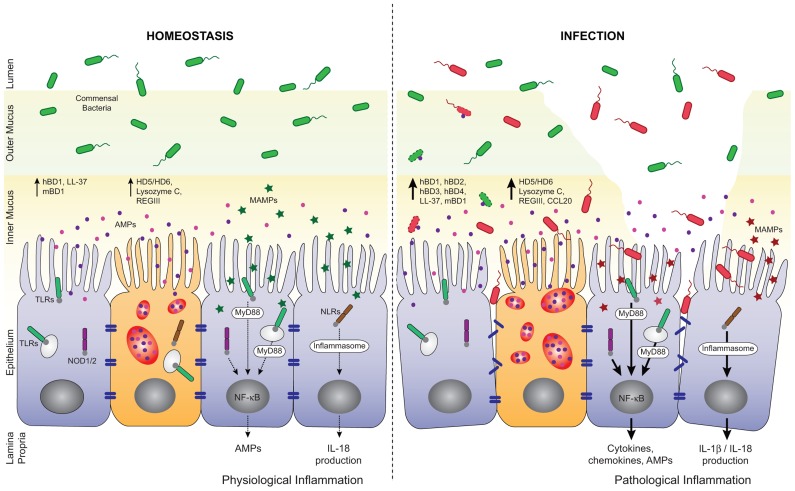
**Regulation of AMP secretion by TLRs and NLRs during homeostasis and infection.** AMPs, mainly secreted by enterocytes and Paneth cells, are found in the inner mucus layer and play important roles in the barrier against enteric pathogens. At the steady state, microbial-associated molecular patterns, (MAMPs, green stars) from commensal bacteria are sensed by pattern recognition receptors (PRRs). They trigger basal AMP and interleukin-18 (IL-18) production through the activation of NF-κB and inflammasome pathways, respectively. This participates in the tuning of host responses toward tolerance and help maintaining intestinal homeostasis. Loss of homeostasis occurs when pathogens breach the mucus layer, enter in contact with IECs and disrupt the epithelial barrier integrity. Pathogens and their products (MAMPs, red stars) are also sensed by PRRs and induce MyD88- or inflammasome-dependent expression of a pro-inflammatory and antimicrobial program. In consequence, high levels of AMPs and cytokines/chemokines are produced that help limit pathogen propagation and contribute to the recruitment of immune cells. hBD, human β-defensin; mBD, mouse β-defensin; HD, human α-defensin.

It is expected that indiscriminate recognition of MAMPs by TLRs and NLRs on the epithelium would trigger an inflammatory response via nuclear factor-κB (NF-κB) activation and subsequent pro-inflammatory cytokine/chemokine and AMP production (Kawai and Akira, 2011; **Figure 2**). However, the epithelium seems to tolerate the presence of commensals. Inflammation only occurs after disruption of the epithelial barrier and activation of TLRs and NLRs by commensals is fundamental for protection against injury (Rakoff-Nahoum et al., 2004; Yeretssian, 2012). TLRs are expressed by IECs with relatively low but inducible levels throughout infection or intestinal inflammation (Otte et al., 2004; Kawai and Akira, 2011). The spatial distribution of TLRs in the epithelium, their cell lineage specificity and functions within the gut were reviewed elsewhere (Abreu, 2010). NLRs are cytosolic PRRs related to disease resistance R proteins in plants and to apoptosis protease activating factor 1 (APAF1; Inohara et al., 1999; Belkhadir et al., 2004). Among the NLRs, NOD1 and NOD2 induce proinflammatory and antimicrobial gene transcription through NF-κB and mitogen-activated protein kinase (MAPK) pathways (Chamaillard et al., 2003; Girardin et al., 2003; Inohara et al., 2003; **Figure 2**). Recent advances in the field have linked several apoptosis effectors such as cIAP1 and cIAP2 (cellular inhibitor of apoptosis proteins 1 and 2) as well as BID (BH3 interacting-domain death agonist) to NOD signaling at the mucosal surface, extending further the mounting parallel between cell death and innate immunity pathways (Bertrand et al., 2009; Yeretssian et al., 2011). NOD1 is constitutively expressed in IECs, while NOD2 expression is confined to Paneth cells in the small intestine. Other members of the NLR family such as NLRC4, NLRP3, NLRP6, and NLRP12 activate macromolecular complexes termed inflammasomes through recruitment and activation of the proinflammatory caspase-1 and subsequent maturation of IL-1β and IL-18 (Yeretssian, 2012; **Figure 2**). The majority of these inflammasome-activating NLRs are expressed in IECs and they contribute in the steady state regulation of the commensal microflora through the induction of a basal secretion of IL-18 by the IECs. Recently, it was reported that deficiencies in the NLR/Caspase-1/IL-18 pathways are associated with a more severe phenotype in mouse models of acute dextran-sulfate sodium (DSS) colitis, possibly mediated, in part, by a defect in microbiota composition and in mucosal tissue repair (Allen et al., 2010, 2012b; Dupaul-Chicoine et al., 2010; Salcedo et al., 2010; Zaki et al., 2010, 2011; Elinav et al., 2011).

Accumulating evidence suggests that TLRs in Paneth cells tightly regulate AMP production via their adaptor molecule MyD88 in the prospect to maintain a healthy gut environment. Transgenic mice expressing a dominant-negative mutant form of MyD88 (dnMyD88) and mice lacking MyD88 had decreased Paneth cell-derived α-defensins (cryptdins), RegIIIβ, RegIIIγ, and RELMβ (Brandl et al., 2007; Rakoff-Nahoum and Medzhitov, 2007; Vaishnava et al., 2008; Gong et al., 2010). Indeed, aged dnMyD88 transgenic mice develop spontaneous intestinal inflammation due to a drop in AMP secretion and continuous bacterial translocation (Gong et al., 2010). AMP production is also inducible in a TLR-dependent manner in response to a wide range of TLR agonists. For instance, *in vivo* stimulation of TLR3 and TLR9 with polyinosinic:polycytidylic acid (poly I:C) and CpG oligodeoxynucleotides (CpG-ODN), respectively, triggers rapid Paneth cell degranulation and release of high quantity of AMPs (Rumio et al., 2012; **Figure 2**). It has been shown that hBD2 is up-regulated in IECs following TLR2, TLR3, and TLR4 engagement and in an NF-κB-dependent manner (Vora et al., 2004; Omagari et al., 2011). Besides, AMP secretion in the gut is not solely exclusive to Paneth cells. It was proposed that hormone producer enteroendocrine cells sense luminal bacterial antigens through TLR4, TLR5, and TLR9, and neutralize intestinal bacteria by releasing chemokines and hBD2 (Palazzo et al., 2007).

NOD-like receptors turn out to act equally as key gatekeepers in the gut and disrupted expression of these receptors is associated with an altered expression of AMPs. Unlike *MyD88*^-/-^ mice, *Nod2*^-/-^ mice present normal levels of RegIIIβ, RegIIIγ, and RELMβ (Abe et al., 2006; Vaishnava et al., 2008; **Figure 2**). However, the expression of α-defensins is significantly reduced in these mice. It was described that *Nod2*^-/-^ mice harbor an increased load of commensal resident bacteria compared to wild-type mice and that they have a diminished ability to prevent intestinal colonization by pathogenic bacteria (Petnicki-Ocwieja et al., 2009; Rehman et al., 2011). Under specific pathogen free conditions, *Nod1*^-/-^Nod2^-/-^ mice had lower RegIIIγ expression compared to their littermate controls (Natividad et al., 2011). These mice were more susceptibility to DSS-induced colitis; a phenotype that was reversed by colonization of the mice with altered Schaedler flora or *Bifidobacterium breve* and that normalized RegIIIγ expression levels. Likewise, NLRP3 and NLRP6 inflammasomes are major regulators of intestinal homeostasis (Elinav et al., 2011; Hirota et al., 2011). In fact, *Nlrp3*^-/-^ mice display altered colonic mBD1 expression and a unique intestinal microbiota compared to wild-type animals (Hirota et al., 2011). *Nlrp6*^-/-^ mice on the other hand show a radical change in microbial community composition prominent to the expansion of colitogenic bacteria and shifting the balance toward a pro-inflammatory configuration that drives spontaneous and induced colitis (Elinav et al., 2011). Microbicidal and chemotactic activities of AMPs require different concentrations of a particular molecule and may be relevant in different situations during infection and disease.

## AMP PRODUCTION IN RESPONSE TO ENTERIC BACTERIAL PATHOGENS

Despite the presence of multilayered innate defense barriers and basal physiological and protective inflammation, enteric bacterial pathogens can efficiently circumvent these barriers, infect and multiply within the gut mucosa. Enteric pathogens such as *Salmonella* spp., *Shigella* spp., *Listeria* spp., *Citrobacter rodentium*, enteropathogenic *Escherichia coli* (EPEC), and enterohemorrhagic *E. coli* (EHEC) subvert the microbiota through highly sophisticated and effective strategies; particularly by diverting AMP production (**Figure 2**). Infection with these pathogens accounts for significant morbidity and mortality worldwide and causes outbreaks in developed countries.

Enteropathogenic *E. coli* and EHEC are human diarrheal pathogens poorly pathogenic in mice (Guttman et al., 2006). *C. rodentium* serves as an excellent surrogate model for studying EPEC and EHEC pathogenesis in mice (Mundy et al., 2005). These bacteria use attaching and effacing (A/E) lesion formation as a major mechanism of tissue targeting and infection. *In vitro* experiments using Caco-2 IECs have shown that EPEC infection promotes a rapid induction of hBD2 and CCL20 as well as other proinflammatory molecules in a type three-secretion system (TTSS)-dependent manner (Khan et al., 2008; **Table 1**). Additionally, HT-29 human colonocytes depleted from NOD2 and infected with *C. rodentium* exhibited diminished levels of hBD2 (LeBlanc et al., 2008). *C. rodentium* as many other enteric pathogens has the ability to takeover host-signaling and modulate innate and adaptive immune responses at epithelial surfaces. Wild-type C57BL/6 mice infected with *C. rodentium* up-regulated mBD1 and mBD3 in the colonic tissue (Simmons et al., 2002). Similarly, enterocytes from mice lacking caspase-12, an endogenous inhibitor of the inflammasome and NF-κB pathways, hyper-produced mBD1 and a subset of chemokines in a NOD2-dependent manner (LeBlanc et al., 2008). Interestingly, *Nod2*^-/-^ mice are more susceptible to *C. rodentium* infection and present a delayed bacterial clearance (Geddes et al., 2011). Moreover, NOD1 and NOD2 have been shown to be crucial for host defense against *C. rodentium* as well as *Salmonella* pathology by triggering an early Th17 response (Geddes et al., 2011). The mouse cathelicidin CRAMP encoded by the gene *Cnlp* also contributes to *C. rodentium* clearance. *Cnlp*^-/-^ mice are more susceptible to *C. rodentium* infection as their colonic epithelial extracts have reduced antimicrobial activity compared to their wild-type littermates (Iimura et al., 2005). Recently, it was described that microbiota transplantation from resistant to susceptible wild-type mice before *C. rodentium* infection caused delayed pathogen colonization and mortality due to IL-22 mediated production of RegIIIβ and RegIIIγ (Willing et al., 2011).

**Table 1:** Human and mouse intestinal antimicrobial peptides and their expression during infection and intestinal inflammation.

Class	Name	Specie	Producing cells	Expression during infection	Reference
α-Defensins	HD-5, HD-6	H.s.	Paneth cells	*Salmonella*↑	Porter et al. (2002), Fahlgren et al. (2003),
				CD↓	Ganz (2003), Wehkamp etal. (2004)
				UC↑	
	Cryptdin	M.m.	Paneth cells	*S. typhimurium*↑	Salzman etal. (2003), Shanahan etal. (2011)
	CRS	M.m.	Paneth cells	–	Shanahan etal. (2011)
β-Defensins	hBD1	H.s.	Enterocytes	*Shigellal*↓	O’Neil et al. (1999), Islam et al. (2001),
				CD↓	Wehkamp etal. (2003)
				UC↓	
	hBD2	H.s.	Enterocytes	EPEC↑	O’Neil etal. (1999), Ogushi etal. (2001),
				*S. typhimurium*↑	Fahlgren et al. (2003), Khan et al. (2008),
				UC↑	Langhorst etal. (2009)
				CD	
	hBD3, hBD4	H.s.	Enterocytes	*Shigella*	O’Neil etal. (1999), Sperandio etal. (2008)
	hBD5, hBD6	H.s.	Enterocytes	–	O’Neil etal. (1999)
	mBD1	M.m	Enterocytes	*C. rodentium*↑	O’Neil etal. (1999), Simmons etal. (2002), Rehman etal. (2011)
	mBD3	M.m.	Enterocytes	*C. rodentium*↑	O’Neil etal. (1999), Simmons etal. (2002),
				DSS↓	Rehman etal. (2011)
	mBD2, mBD4,	M.m.	Enterocytes	–	O’Neil etal. (1999)
	mBD5				
Cathelicidins	LL-37/hCAP18	H.s.	lECs	*Shigella*	Islam et al. (2001), Hase etal. (2002),
				UC↑	Schauber etal. (2006)
	CRAMP	M.m.	IECs	DSS↑	Koon etal. (2011), Allen etal. (2012a)
				*L. monocytogenes↑*	
Other AMPs	RegIIIα or HIP/PAP	H.s.	Paneth cells	–	Lasserre etal. (1999), Vaishnava etal. (2008)
	Reglllβ	M.m.	Paneth cells	*C. rodentium*↑	Willing et al. (2011)
	Regllly	M.m.	Paneth cells	*Salmonella*↑	Lasserre etal. (1999), Vaishnava etal. (2008),
				*C. rodentium*↑	Godinez etal. (2009)
	sPLA2	H.s.	Paneth cells	–	Qu etal. (1996)
	CCL20/MIP-3α	H.s./M.m.	lECs	EPEC↑	Kaser etal. (2004), Teramoto etal. (2005),
				UC↑	Katchar etal. (2007), Khan etal. (2008),
				CD↑	Godinez etal. (2009)
				DSS/TNBS	
	Lysozyme C	H.s.	Paneth cells	–	Ganz (2004)
	Lysozyme C type P	M.m.	Paneth cells	*S. typhimuriuml*↓	Cross et al. (1988), Salzman etal. (2003),
				UC↑	Ganz (2004)
	BPI	M.m.	IECs	UC↑	Schinke etal. (2004)
				CD↑	
	BPI	H.s.	IECs	–	Canny etal. (2002)
	ANG4	M.m.	Paneth cells	–	Hooper etal. (2003)
	RELMβ	H.s./M.m.	Goblet cells	–	Artis etal. (2004), Hogan etal. (2006)

*H.s., Homo sapiens; M.m., Mus musculus.*

*Salmonella enterica* serovar *typhimurium* (*S. typhimurium*) is another enteropathogen that causes acute systemic inflammation by using virulence factors to invade the intestinal epithelium (Broz et al., 2012). During infection, *S. typhimurium* competes with the microbiota, changes the bacterial community structure and outgrows. It was described that *S. typhimurium* induces the expression of hBD2, but not the constitutively existing hBD1, in human fetal intestinal xenografts as well as HT-29 and Caco-2 cell lines in a FliC (flagella filament protein)-dependent manner (Bonham et al., 1999; Ogushi et al., 2001). Mature HD-5 has potent bactericidal activities against *S. typhimurium* and *E. coli* infections (Wehkamp et al., 2005; Ishikawa et al., 2010). MMP7 deficient mice lack mature cryptdins; they have an impaired intestinal clearance of *S. typhimurium* and *Shigella*, and thus greater susceptibility to the infection (Wilson et al., 1999; Atarashi et al., 2011). Actually, HD-5 transgenic mice are protected against *S. typhimurium* infection (Salzman et al., 2003). In a mouse model of *Salmonella* colitis, RegIIIβ and RegIIIγ expression is markedly increased (Godinez et al., 2009). Unlike other pathogens such as *Listeria monocytogenes*, *Salmonella* takes advantage of RegIIIβ/γ secretion, subverts the innate defense and grows over the microbiota (Godinez et al., 2009; Stelter et al., 2011). Infection of wild-type mice with *S. typhimurium* also decreases the expression of cryptdins and lysozyme. However, the use of heat killed *S. typhimurium* that lacks the *Salmonella* pathogenicity island 1 (SPI-1) TTSS and heat killed *L. monocytogenes* did not affect AMP production (Salzman et al., 2003).

*Shigella*, an enteric pathogen closely related to *Salmonella*, was found to switch-off LL-37 and hBD1 expression during infection probably facilitating its invasion and increasing its virulence (Islam et al., 2001). Similarly, infection of polarized human IECs with *S. flexneri* suppressed specifically the transcription of hBD3 (Sperandio et al., 2008). Interestingly, butyrate was found to enhance LL-37 expression in IECs and treatment of mice with butyrate decreased the severity of experimental Shigellosis (Raqib et al., 2006). Several AMPs exert potent antimicrobial activity against *L. monocytogenes* including LL-37 and CRAMP (Turner et al., 1998; Menard et al., 2008). CRAMP was constitutively expressed in the IECs of mice early after birth conferring gut homeostasis and mediating protection against *L. monocytogenes* infection (Menard et al., 2008).

There is an intricate yet mutualistic relationship between the host and its microorganisms within the GI tract. Consequently, genetic or acquired alterations in the functions of the mucosal immune barriers may profoundly impact homeostasis and result in chronic inflammatory diseases.

## AMPs AND INFLAMMATORY BOWEL DISEASE

Disturbance of intestinal homeostasis results in an altered composition of the intestinal microbiota or dysbiosis, a hallmark of IBDs. Crohn’s disease (CD) and ulcerative colitis (UC), the two major forms of IBD, are characterized by chronic relapsing inflammation of the digestive tract. IBD is a multifactorial disease caused by complex interaction of genetic, microbial, and immunological factors. Linkage analysis, candidate gene approaches, and genome-wide association studies have identified several risk genes for IBD (Barrett et al., 2008; Franke et al., 2010), many of which are involved in innate immune recognition of bacteria [e.g., NOD2 (Hugot et al., 2001) and NLRP3 (Villani et al., 2009)] or processing and elimination of bacteria through the autophagy pathway [e.g., IRGM (Singh et al., 2006) and ATG16L1 (Rioux et al., 2007)]. There is a paramount role of immune effectors such as PRRs and AMPs in the intestine. Thus, it is relevant to comprehend whether AMP secretion is impaired during IBD.

Decreased Paneth cell production of HD-5 and HD-6 was observed in surgical specimens and biopsies from CD patients (Wehkamp et al., 2005; **Table 1**). Nevertheless, it is still controversial whether CD-associated *NOD2* mutations contribute to abnormal defensin secretion (Wehkamp et al., 2004; Simms et al., 2008). In fact, CD patients have an impaired processing and maturation of HD-5 and administration of exogenous mature HD-5 to DSS-treated mice significantly improved the colitis outcome (Elphick et al., 2008; Ishikawa et al., 2010). Colonic epithelial cells from UC patients exhibited high mRNA levels of lysozyme, HD-5, HD-6, and hBD2 compared to cells from healthy controls (Fahlgren et al., 2003). Similarly, hBD2 expression was reported in patients with irritable bowel syndrome and in Celiac disease (Langhorst et al., 2009; Vordenbaumen et al., 2010). In mice, mBD3 levels are induced following acute DSS-colitis (Rahman et al., 2011). Moreover, cryptdin- and MMP7-deficiency render mice more susceptible to DSS-induced colitis (Wilson et al., 1999). hBD1 is the only β-defensin constitutively expressed in IECs, however its expression is reduced in CD and UC patients (Wehkamp et al., 2003). Genetic variations in the gene encoding hBD1 were found to be associated with the risk for CD (Kocsis et al., 2008). LL-37 expression is increased in inflamed and non-inflamed mucosa in patients suffering from UC but not in CD (Schauber et al., 2006). Likewise, CRAMP is induced during intestinal inflammation and *Cnlp*^-/-^ mice are protected from DSS-colitis following administration of a synthetic cathelicidin (Koon et al., 2011; Allen et al., 2012a). Increasing evidence show that polymorphisms in BPI are linked to IBD susceptibility (Akin et al., 2011). BPI levels are greater in biopsies from UC and CD patients and the protein is a frequent target antigen of autoantibodies in the sera of IBD patients (Monajemi et al., 1996; Haapamaki et al., 1999; Schinke et al., 2004). Although CCL20 is produced in normal and inflamed colon tissues, its expression is significantly higher in biopsies from UC and CD patients as well as in peripheral blood mononuclear cells from UC patients (Kaser et al., 2004; Fujimaki et al., 2005). Finally, CCL20 is similarly increased in mice following DSS- and TNBS-induced colitis; hence neutralizing its expression with antibodies is sufficient to reduce the inflammatory outcome (Teramoto et al., 2005; Katchar et al., 2007).

## CONCLUSION

Over the past decade our knowledge of the AMPs as immune effectors at the intestinal epithelial interface has increased exponentially. Their importance in health and disease has only recently been appreciated and our understanding of their regulatory mechanisms remains at an early stage. AMPs possess pleiotropic functions to not only eradicate microbes in the GI tract but also control homeostasis and host immunity. AMPs promote innate and adaptive immune responses to invading microbes through the recruitment and activation of leukocytes via their interaction with chemokine and formyl peptide receptors. Dysbiosis and alterations in intestinal microbiota are essential for the development of IBD pathophysiology and emerging evidence points to a possible central role for AMPs in determining the composition of these microbes. Several mechanisms have been hypothesized to explain how AMPs modulate antimicrobial immunity in the gut barrier. However, many more questions remain to be addressed. How AMPs interact with the commensals and help maintaining a functional gut immune barrier? How the presence of the microbiota is affecting the production of these effector molecules? Are PRRs (TLRs and NLRs) the sole receptors to induce their expression? Another open question is how the chemotactic properties of AMPs play a role in the response against microbial infections and how they bridge innate and adaptive immunity? Better understanding of the mechanisms is expected to open new AMP-based therapeutic avenues to battle infections and strategies for IBDs. Future studies will focus on the regulatory mechanisms that maintain appropriate expression of these antimicrobial factors, and will provide new insights into the role of AMPs in intestinal homeostasis and in the composition of the intestinal microbiota during health and disease.

## Conflict of Interest Statement

The authors declare that the research was conducted in the absence of any commercial or financial relationships that could be construed as a potential conflict of interest.
